# 197. Evaluation of Risk Factors and Outcomes Associated with Daptomycin-nonsusceptible *Staphylococcus aureus* Bacteremia

**DOI:** 10.1093/ofid/ofad500.270

**Published:** 2023-11-27

**Authors:** Zachary W Hanna, George J Alangaden, Marcus Zervos, Geehan Suleyman

**Affiliations:** Henry Ford Health, Detroit, Michigan; Henry Ford Health, Detroit, Michigan; Henry Ford Hospital, Detroit, Michigan; Henry Ford Health, Detroit, Michigan

## Abstract

**Background:**

*Staphylococcus aureus* (SA) is a major cause of hospital-associated infections in the US with high morbidity and mortality. With SA developing resistance to many first-line antibiotics, daptomycin (DAP) has become a critical agent for SA therapy. However, increasing use of DAP has resulted in emergence of DAP-nonsusceptible (DNS) SA strains. We aim to elucidate risk factors associated with DNS SA and compare outcomes between patients with DAP-susceptible (DS) and DNS SA bacteremia (SAB).

**Methods:**

Retrospective cohort analysis was performed on patients with DNS (cases) and DS (controls) SAB admitted to Henry Ford Health between 9/2005 and 3/2023. Patients with persistent ( >7 days of positive blood cultures) DS SAB were used as controls. Demographic, risk factors, clinical characteristics, and outcomes were evaluated. Primary outcomes were 30-day relapse or progression, readmission and mortality; secondary endpoint was 90-day mortality.

**Results:**

A total of 122 patients included 59 (48%) cases and 63 (52%) controls. The majority were male (56.6%) with median age of 59. The study population had a high burden of comorbidities as outlined in the **Table**; central venous catheter use was significantly more common among cases (p=0.049). History of MRSA infection (p< 0.001) and prior hospitalization (p< 0.001) within 1-year, and antibiotic (p=0.017) use, particularly vancomycin (p=0.011), within 90 days were associated with DNS SA. Primary source of infection and infectious complications were not significantly different among cases and controls. There was no significant difference in outcomes between the two groups. Although not statistically significant, 90-mortality was higher in the DNS group (p=0.075).

**Figure:**
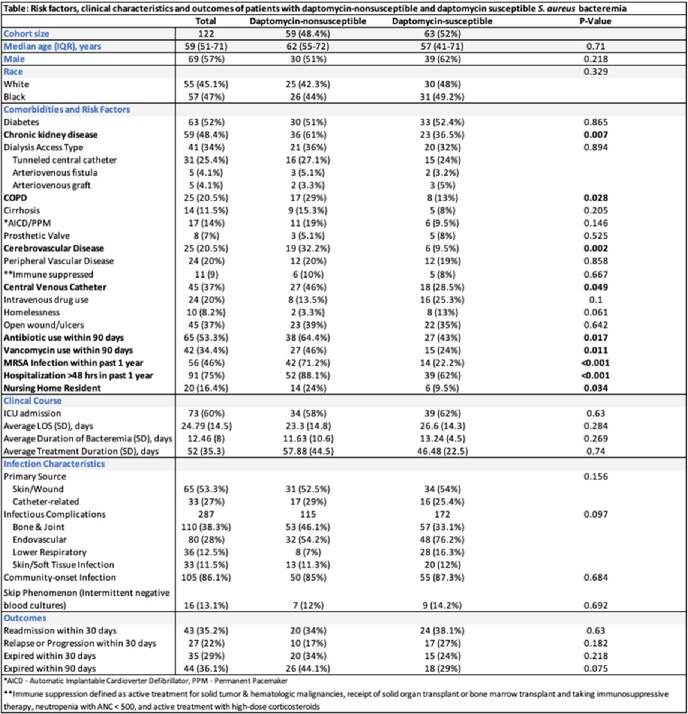

**Conclusion:**

Our study highlights risk factors for DNS SA, including recent vancomycin use, and prior hospitalization and MRSA infection within 1 year. While there was no significant difference in outcomes between DNS and DS SAB, overall mortality was high. These findings highlight the need for continued surveillance of DNS SA and careful consideration of risk factors when selecting antimicrobial agents for complicated SA infections. Further studies are needed to identify potential mechanisms of vancomycin cross-resistance in DNS SA.

**Disclosures:**

**Marcus Zervos, MD**, Contrafect: Advisor/Consultant|GSK: Grant/Research Support|Johnson and Johnson: Grant/Research Support|Pfizer: Grant/Research Support

